# Dependency Structures in Differentially Coded Cardiovascular Time Series

**DOI:** 10.1155/2017/2082351

**Published:** 2017-01-03

**Authors:** Tatjana Tasic, Sladjana Jovanovic, Omer Mohamoud, Tamara Skoric, Nina Japundzic-Zigon, Dragana Bajic

**Affiliations:** ^1^School of Medicine, Laboratory for Cardiovascular Pharmacology, University of Belgrade, Dr. Subotica 1, 11000 Belgrade, Serbia; ^2^Telecom Serbia, Network and Services Planning and Development Function, Bulevar Umetnosti 16a, 11070 Novi Beograd, Serbia; ^3^Faculty of Technical Sciences, Center of Excellence CEVAS, University of Novi Sad, Trg Dositeja Obradovica 6, 21000 Novi Sad, Serbia

## Abstract

*Objectives*. This paper analyses temporal dependency in the time series recorded from aging rats, the healthy ones and those with early developed hypertension. The aim is to explore effects of age and hypertension on mutual sample relationship along the time axis.* Methods*. A copula method is applied to raw and to differentially coded signals. The latter ones were additionally binary encoded for a joint conditional entropy application. The signals were recorded from freely moving male Wistar rats and from spontaneous hypertensive rats, aged 3 months and 12 months.* Results*. The highest level of comonotonic behavior of pulse interval with respect to systolic blood pressure is observed at time lags *τ* = 0, 3, and 4, while a strong counter-monotonic behavior occurs at time lags *τ* = 1 and 2.* Conclusion*. Dynamic range of aging rats is considerably reduced in hypertensive groups. Conditional entropy of systolic blood pressure signal, compared to unconditional, shows an increased level of discrepancy, except for a time lag 1, where the equality is preserved in spite of the memory of differential coder. The antiparallel streams play an important role at single beat time lag.

## 1. Introduction

Interaction of blood pressure (BP) and pulse interval (PI) are complex, governed by numerous homeostatic mechanisms, including the autonomic nervous system [1–3]. Alterations in their functioning either initiate or worsen cardiovascular diseases [4–6]. As a main blood pressure corrector, baroreflex is a subject of numerous studies. A range of methods for estimating its parameters has been developed, both in time domain [7–9] and in frequency domain [10, 11]. Other approaches include the models based on information domain and on nonlinear nature of the systolic BP (SBP) and heart period interactions [12–16]. The comparative analysis is abundant as well, for example, [6, 17]. Besides the baroreflex as a feedback pathway, SBP-PI loop also includes a mechanical feedforward pathway, as PI influences SBP via Frank-Starling law and diastolic runoff [18].

A time delay (lag) of pulse interval (PI) with respect to SBP was included into the baroreflex studies via cross-correlation baroreflex sensitivity (*x*BRS), where the cross-correlation is applied for assessing the time delay that corresponds to the maximum SBP-PI interaction [9]. This lag is actually a delay of PI response with respect to changes in SBP, caused by the signal propagation, as well as processing in the autonomic nervous system. This delay also presents an important clinical marker [6, 19]. A longer delay indicates a weaker response of the parasympathetic and stronger response of sympathetic nervous system and vice versa. It is changeable according to the physiological state; for example, it is longer in standing than in lying position and it increases with the increase of heart rate and age [20]. Time delay is also affected by a reduction of baroreflex sensitivity, heart failure [21], and syncope [22].

One of the most widely used techniques for studying the spontaneous BRR without pharmacological or mechanical interventions is the sequence method [19, 20]. Sequences are the streams of consecutive beats in which progressive increases (or decreases) in systolic blood pressure (a SBP ramp) are followed by progressive increases (or decreases) in pulse interval (a PI ramp), delayed by a time lag that heavily depends on species [21–29]. The ramps and streams in physiological time series can be a consequence of physiological interactions and of mere random occurrence. Short random streams may be indistinguishable from the physiological ones due to the large coefficient of correlation (a consequence of shortness). Long streams, on the other hand, are characteristics of real physiological data only, since their number in random time series is negligible [30]. For this reason, only the streams of length that surpass a predefined threshold, usually three or four beats, are considered as “sequences” [17]. So, to avoid the ambiguity, the term “sequence” would be reserved for a stream with a length that exceeds the threshold.

This paper analyses the level of temporal dependency in time series recorded from aging rats: the healthy ones and the ones with early developed hypertension. The aim is to find a time span (time lag) along which a change in one signal sample affects the changes of other samples from the same or from the related time series and to explore whether the increased age and hypertension affect the mutual sample relationship. Among the tools that measure statistical dependency at signal level investigated in [31] (e.g., Pearson's product-moment correlation that measures linear relationship, Spearman's correlation that measures the monotonic relationships, and Kendall's correlation that reflects the number of concordances and discordances in time series, as well as the classical correlation) we opted to use copula, and among the numerous copula families we opted for the Frank copula, since it was shown to be well suited to cardiovascular time series [31]; it distinguishes comonotonic and counter-monotonic behavior in bivariate signals. The analysis is applied to the source signals and to the differentially coded signals that process the real numbers. Novel applications require on-line analysis in battery-operated devices, implying computationally more efficient procedures. It brings binary operations to the fore, so we applied binary conditional entropy as well. Application range include crowdsensing [32], as well as self-monitoring during the exercise.

## 2. Materials and Methods

### 2.1. Experimental Protocol and Signal Acquisition

All experimental protocols were approved by the Faculty of Medicine University of Belgrade Experimental Animals' Ethics Committee. All procedures conformed to EEC Directive 86/609 and the School of Medicine University of Belgrade Guidelines on Animal Experimentation.

#### 2.1.1. Animals

Experiments were performed in 3- and 12-week-old male Wistar normotensive and spontaneous hypertensive (SHR) rats, weighing 260–400 g. Total number of rats was *n* = 24. Animals were equipped with a right femoral artery catheter for blood pressure recording. Rats were kept in Plexiglas cages (21 cm × 37 cm × 19 cm) under controlled laboratory conditions (temperature 22 ± 1°C, humidity of 65 ± 1%, 7:00 h–19:00 h light-dark cycle) with tap water and pelleted food available ad libitum. The number of animals per experimental group (6) was calculated using software “Power and Sample Size Calculations” for a given power 90% and type I error probability of 0.05 freely downloadable at http://ps-power-and-sample-size-calculation.software.informer.com/.

#### 2.1.2. Surgery

Under combined xylazine 2% (10 mg/kg i.p.) and ketamine 10% (90 mg/kg; i.p.) anesthesia, a polyethylene catheter (OD = 0.90, ID = 0.58, Smiths Medical International Ltd., Kent, UK) prefilled with heparinized saline (50 IU/mL) was inserted in the right femoral artery and tunneled subcutaneously between scapulae for BP recording. Perioperatively rats received gentamicin (25 mg/kg, i.m.) to prevent infection and carprofen (5 mg/kg, s.c.) for pain relief. The sutures in the inguinal and interscapular regions were sprayed with topical antibiotics. After surgery, each rat was housed individually in Plexiglas cages (30 cm × 30 cm × 30 cm) under standard laboratory conditions and left to recover for two days.

#### 2.1.3. Cardiovascular Signal Acquisition and Preprocessing

Arterial blood pressure was recorded for 30 minutes on polygraph (Hugo Sachs Electronics D79232, Freiburg, Germany) and digitalized at 1000 Hz. Systolic (SBP) and diastolic BP (DBP) and pulse interval (PI) were derived from the arterial pulse pressure as maximum, minimum, and interbeat interval, respectively. The derived signals were inspected for misdetections and artifacts and manually corrected. For each registration period, mean value of SBP, DBP, and PI was calculated and again averaged for the whole experimental group. Other usual analytical methods include Poincaré Plots (PPlots) and cross-approximate entropy (X*ApEn*). PPlot is primarily a visual method; its spotted images correspond to the 2D joint probability distribution function. The PPlot quantitative parameters are standard deviations of signals $x_{1i}=\sqrt{2^{-1}}\cdot (PI_{i+1}-PI_{i})$ and $x_{2i}=\sqrt{2^{-1}}\cdot (PI_{i+1}+PI_{i}),\;\;i=1,\ldots ,N-1$, describing short and long term variability of the pulse interval time series [33]:

(1) SD1CPI0−CPI1=EPIi−EPI2−EPIi−EPI·PIi+1−EPI,SD2CPI0+CPI1=EPIi−EPI2+EPIi−EPI·PIi+1−EPI,

where *E*{ } is an expectation operator, *C*( ) is a covariance function, *N* is the time series length, SD is standard deviation, and the subscript in *E*{PI} is omitted since the signals are assumed to be wide sense stationary (WSS). X*ApEn* [34] is a classical static measure of the mutual interreaction of parallel time series. In brief, X*ApEn* procedure divides each time series into *N* − *m* + 1 overlapping vectors of length *m*. A selected vector from the first series is compared to each one of the *N* − *m* + 1 vectors from the second series, to estimate the probability that their absolute distance is below the specified threshold. It is repeated for each one of the *N* − *m* + 1 vectors from the first series, and the logarithms of the estimated probabilities are averaged (the first average). Then the procedure is repeated for the vectors of length *m* + 1. The obtained second average is subtracted from the first one, yielding X*ApEn* estimate [34].

As a control, pseudorandom and randomized signals were implemented. Pseudorandom signals include series of independent and identically distributed (i.i.d.) random variables with normal and uniform distribution. Randomized signals include surrogate data series [35]. Isodistributional surrogates randomize the temporal order of the observed time series and destroys the sample dependency but preserve the signal distributional function. Isospectral surrogates operate in transform domain, either randomizing the existing signal phases, or substituting them with pseudorandom i.i.d. phase samples with uniform distribution. In both cases, the power spectral density remains unchanged and, according to the Wiener-Khinchin theorem, the same applies to the autocorrelation function, so the intersample connections are preserved [36].

#### 2.1.4. Drugs

Ketamine, xylazine, and carprofen (Rimadyl®) as well as the combination of embutramide, mebezonium, and tetracaine (T61®) injections were purchased from MarloFarma (Belgrade, RS). Gentamicin injection and bacitracin neomycin spray (Bivacyn®) were purchased from Hemofarm (Vršac, RS).

#### 2.1.5. Statistical Analysis

Results are shown as mean ± standard error of the mean. Statistical comparison between experimental groups was done using Mann–Whitney test in GraphPad Prism 4 software (GraphPad Software Inc., San Diego, CA, USA). The level of significance was set at *p* < 0.05.

### 2.2. Analytical Methods

The level of dependence inherent to SBP and PI time series is assessed in two ways: using copula analysis of original and differentially coded data and estimating mutual uncertainty using computationally efficient binary conditional entropy, applied to binary differentially coded time series. The inclusion of the second analysis is initiated by the increasing number of battery-operated wearable monitoring devices.

A copula is a mathematical concept that decomposes a multivariate (in this case: bivariate) distribution functions into its univariate marginals, measuring the global statistical dependency among the components. Its release in [37] initiated an extensive implementation within the various fields, but the applications in biomedical studies are rare, including imaging-based diagnostic classifiers for neuropsychiatric disorders [38], the aortic regurgitation [39], and a drug sensitivity prediction [40]. The possibility of applying a copula for cardiovascular signals is pointed out in [41], while its pharmacological validation is performed in [31]. In brief, observing a set of *NV* variables (RV) *x*_*i*_ with a joint distribution function *J*(*x*_1_, *x*_2_,…, *x*_*NV*_) and with respective marginal distribution functions *F*_1_, *F*_2_,…, *F*_*NV*_, a new set of variables *u*_*i*_, uniformly distributed on [0,1]^*NV*^ [42, 43], can be derived as *u*_*i*_ = *F*_*i*_(*x*_*i*_), *i* = 1,…, *NV*. The corresponding copula is defined as

(2) Cu1,u2,…,uNV=JF1−1u1,F2−1u2,…,FNV−1uNV.

or

(3) Jx1,x2,…,xNV=CF1x1,F2x2,…,FNVxNV.

It was shown that Frank copula is the most suitable for cardiovascular signals [31]: it is unbounded and symmetric with value zero in absence of dependence, its sensitivity for SBP-PI signal is the best, and, for a bivariate case, it permits modelling both comonotonic and counter-monotonic dependence. The Frank copula distribution is given by the following relation:

(4) CFu1,u2,…,uN=−θ−1·log⁡1+∏i=1Ne−θ·ui−1e−θ−1N−1.

The copula parameter *θ* shows the level of statistical dependence and in Frank case it is set to zero if the variables are completely independent.

**Figure 1:**
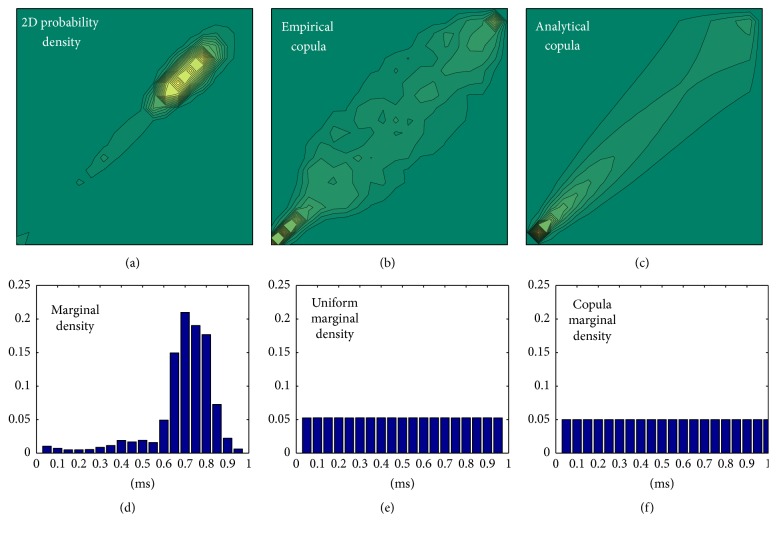
Visualization of the copula process. (a) Estimated joint pulse interval density function with time lag 1 (it corresponds to PPlot); (b) estimated copula density showing the level of the dependency structure in [0,1]^2^ plane; (c) the best fit theoretical copula (Clayton, *θ* = 4.2617, 20 bins). (d, e, f) 1D marginal densities; (d) PI time series; (e) and (f) transformed and calculated uniform density.

**Figure 2:**
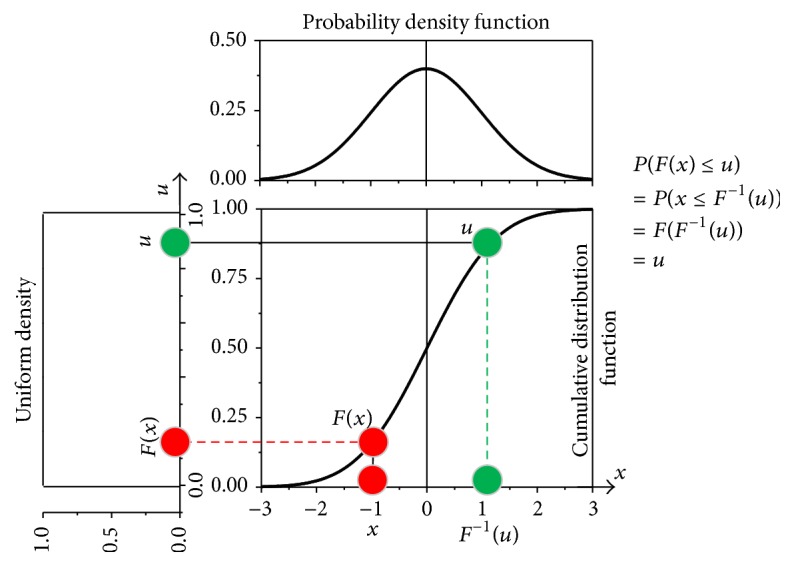
Transformation of a random variable *x* with an arbitrary distribution function *F*(*x*) into a random variable *u* with uniform distribution using *F*(*x*) for transform. The relationship *x* ≤ *F* ^−1^(*u*) along the *x*-axis and *F*(*x*) ≤ *u* along the *u*-axis are unaltered by *F*(*x*) transform, so *P*(*F*(*x*) ≤ *u*) = *u*, which holds for the uniform distribution with RV defined on [0,1].

The copula concept is clarified considering a simple example of two (*NV* = 2) jointly observed time series: pulse interval PI_*i*_ as the first one and a beat delayed counterpart PI_*i*+1_ as the second time series, *i* = 1,…, *N* − 1. Then both the joint empirical probability density function (pdf) and the corresponding empirical marginal pdfs are estimated and shown in Figures 1(a) and 1(d), respectively. The second step in copula procedure is to apply the theory of inverse transform methods [44]. In brief, a random variable (RV) *x* with arbitrary distribution can be transformed into a RV with uniform distribution *u*, using its own distribution function *F*(*x*) for transformation, as explained in Figure 2. Such a transformation yields an empirical copula density function, shown in Figures 1(b) and 1(e): the transform has eliminated the marginal distributions, so only the dependency structure is preserved, revealing that in this example the tail (corner) samples are the ones that exhibit the maximal dependency and not the samples with the most frequent values. The last step of the procedure is to find an analytical copula that is the closest to the obtained empirical one. After choosing the copula family, the analytical copulas for a range of parameter *θ* are generated, and the one that is the closest in a maximum likelihood sense to the empirical one is chosen as a representative copula. This copula density, as well as its uniform marginal, is shown in Figures 1(c) and 1(f).

**Figure 3:**
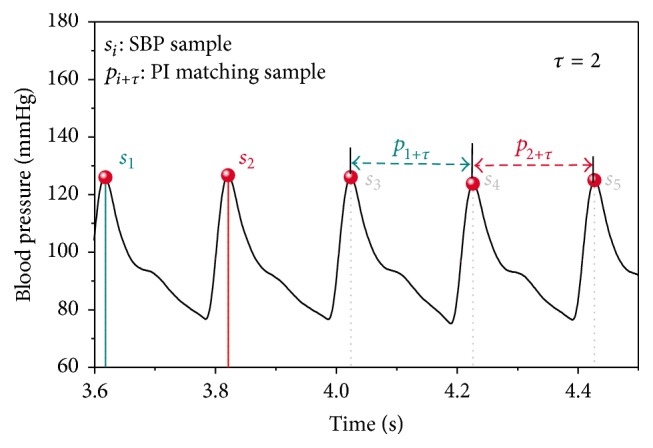
Blood pressure waveform (black line), SBP signal samples (red dots), and PI signal samples at the time lag *τ* = 2; the matching *s*_*i*_-*p*_*i*+*τ*_ signal sample pairs are denoted using the same color.

The joint 2D density in Figure 1(a) visually corresponds to PPlot. Indeed, both techniques start from the same visual presentations. Their further development is different: PPlot is devoted to the short and long term signal variability, expressed through the respective standard deviations SD1 and SD2; copula shows the level of statistical dependence, expressed through the copula parameter *θ*. The copula in this study is applied to bivariate data, to the SBP and PI time series. A level of freedom is a time lag *τ* of PI samples *p*_*i*_, *i* = 1,…, *N*, with respect to SBP samples *s*_*i*_, *i* = 1,…, *N*, so the empirical joint distribution function *J* is estimated over the delayed sample pairs: *s*_*i*_-*p*_*i*+*τ*_, *i* = 1,…, *N* − *τ*, as shown in Figure 3.

The estimated copula density shows a structure of mutual relationship of the SBP and PI time series, that is, the regions where the signal dependency is the strongest. A fitting procedure quantifies the overall dependency level, reducing the copula to a static single value *θ*. But if the time series comprise sufficient amount of data, a dynamic tracking can be performed as well. Time series can be partitioned into the overlapping segments of size *d*, and for each segment a copula dependency parameter *θ* can be evaluated. A series of adjacent *θ* values show the dynamic changes of dependency parameter in time that can be associated with the behavior of the observed subject. Typical segment lengths are 300 to 500 samples, while the overlapping level of adjacent segments is typically *d*/10.

Copula can be applied to the differentially coded signals as well. The differentially coded SBP signal *s*_*i*_ and PI signal *p*_*i*_, *i* = 1,…, *N*, are expressed as

(5) $$\begin{array}{c} xD_{i}=x_{i+1}-x_{i},\;i=1,\ldots ,N-1,\;\;x\in \left\{s,p\right\}. \end{array}$$

In applications where power and processor resources are limited, it is more appropriate to work with binary signals. Binary differentially coded counterparts of the signals from (5) are expressed as

(6) $$\begin{array}{c} xB_{i}=\left\{\begin{array}{cc} 0 & xD_{i}\leq 0\\ 1 & xD_{i}>0, \end{array}\right.\;i=1,\ldots ,N-1,\;\;x\in \left\{s,p\right\}. \end{array}$$

Copulas cannot be applied to the time series transformed into a binary form, but the similar goal can be achieved by unconditional and conditional entropy of a single time series (*H*(**x** **B**)  and  *H*(**x** **B**∣**x** **B**)) and of the joint time series (*H*(**s** **B**, **p** **B**)  and  *H*(**s** **B**, **p** **B**∣**s** **B**, **p** **B**)) as follows: 
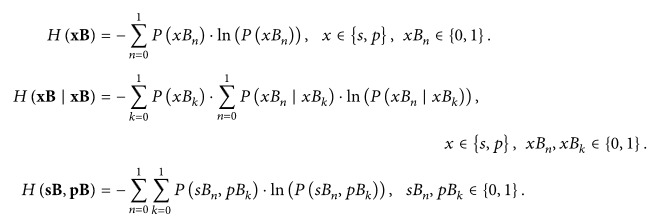
(7)

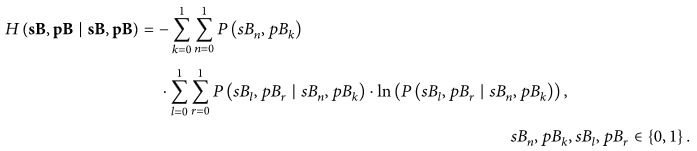
(8)


**Figure 4:**
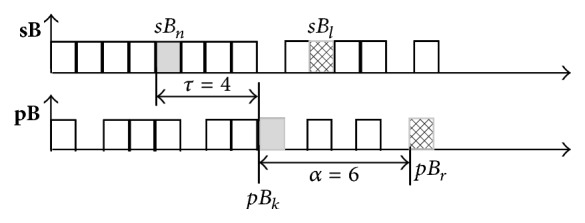
Levels of freedom for mutual positions of binary symbols in (7) and (8): *τ* is time lag between the bit *sB*_*n*_ from SBP and its delayed counterpart *pB*_*k*_ from PI series; *α* is time lag between the bits *sB*_*n*_ and *sB*_*l*_ (*pB*_*k*_ and *pB*_*r*_) within the SBP (PI) time series.

In the above equations, *P*(*x*), *P*(*x*, *y*), and *P*(*x*∣*y*) denote a probability, a joint probability, and a conditional probability. Since entropy, as a rule, is a relative measure, conditional entropy (lower parts of (7) and (8)) is usually presented in percentage of its unconditional counterpart (upper part of the same equations). The relationship between the binary symbols in (7) and (8) is shown in Figure 4. 

There are two levels of freedom for estimating the joint entropy: time lag *τ* between the pairs of bits from SBP and PI time series and time lag *α* within the bits of that belong to the same time series (either SBP or PI). It should be noted that indices *n*, *k*, *l*, and *r* in Figure 4 correspond to (7) and (8), that is, to the binary symbols and not to the time axis. For example, if the bit *sB*_*n*_ in Figure 4 is the *i*th bit along the time axis, then the bits *pB*_*k*_, *sB*_*l*_, and *pB*_*r*_ would be at the positions *i* + *τ*, *i* + *α*, and *i* + *τ* + *α*.

**Figure 5:**
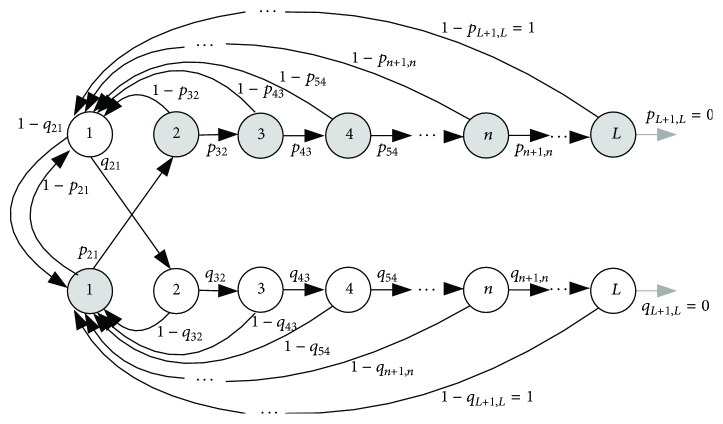
A two-branch counter; states correspond to the successive positive and negative differential signal (5), that is, to the successive increasing and decreasing amplitudes.

Figure 5 presents a two-branch counter [30] with states corresponding to the successive positive signal differences, that is, successive increasing signal amplitudes (branch with gray states) and successive decreasing signal amplitudes (branch with white states). If the counter is in the state denoted “*k*,” it means that *k* signal differences of the same sign have already occurred in a row. It is in a form of a finite ergodic Markov chain and it models the increasing and decreasing ramps of differentially encoded signal samples.

If a ramp in SBP signal is followed by a ramp in PI signal at a particular time lag *τ* and if the ramps comprise either increasing or decreasing differences, such ramps form a “parallel stream.” Similarly, antiparallel stream may be defined as an increasing SBP ramp followed by decreasing PI ramp at a time lag *τ* (and vice versa). The occurrence of parallel and antiparallel streams will be of importance for dynamic tracking and explanation of copula parameters.

The model shown in Figure 5, aided with the theory of Markov chains, enables analytical evaluation of parameters, such as the exact number of ramps and streams in i.i.d. random data, as well as the state transition probabilities evaluation:

(9) NRAMPn=N−1·n+12+nn+3!,NSEQn=N−1·2·n+12·n+32−n+22n+3!2,pn+1,nn=n+2n+1·n+3.

The detailed mathematical derivation of the expressions (9) is given in [30].

## 3. Results and Discussion

### 3.1. Static Results

The results of the conventional analyses are presented in Table 1. While the PI statistics and the corresponding Poincaré Plot measures SD1 and SD2 were statistically the same in all four groups, blood pressure, both SBP and DBP, was high in spontaneous hypertensive (SHR) rats, and, with their increasing age, pressure significantly increased. X*ApEn* could not make any distinction between the observed groups. Further on, although X*ApEn* is frequently used in assessing the level of interrelations of time series, it has no possibility to observe if one time series is delayed with respect to another: X*ApEn*, by definition, compares all possible combinations of *m*-sized partitions of both time series, producing always the same result, regardless of time lag *τ*.

**Figure 6:**
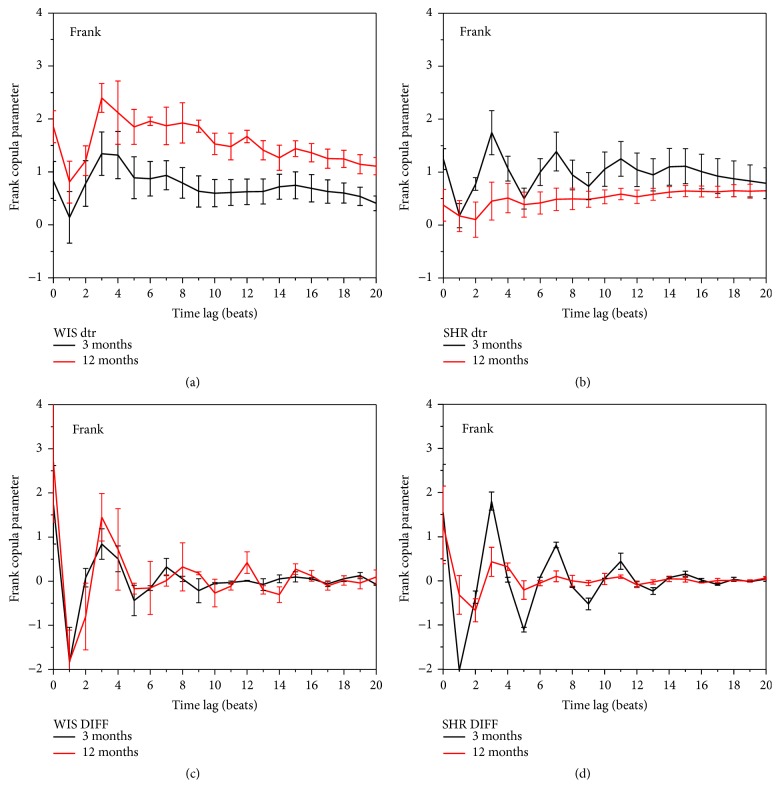
A static copula dependency measure estimated from the original signals with trend removed and from the differentially coded signals ((a–d), resp.); (a, c) correspond to Wistar normotensive rats; (b, d) correspond to spontaneous hypertensive rats.

Copula, however, can take the time lag *τ* between the observed time series into account.  Figure 6 presents a static copula measure, calculated over 4000 SBP-PI pairs, as a function of a particular time lag *τ*. (a, b) show the dependency estimated from the original detrended [45] signals, while (c, d) show dependency estimated from the differentially coded signals (5). Copula parameters derived from differentially coded signals show that the statistical dependency induced by signal changes are consistent with time lags in all four experimental groups: the highest dependence is observed at lags *τ* = 0 and *τ* = 3, while a strong negative dependency occurs at time lag *τ* = 1. While the dependency of SBP-PI changes remained intact in healthy rats with increased age (c), small values of *θ*, almost close to zero, in aging hypertensive rats show loosening the SBP-PI connections (b), also emphasized by decreased dynamic range dependency level of SBP-PI changes (d).

**Figure 7:**
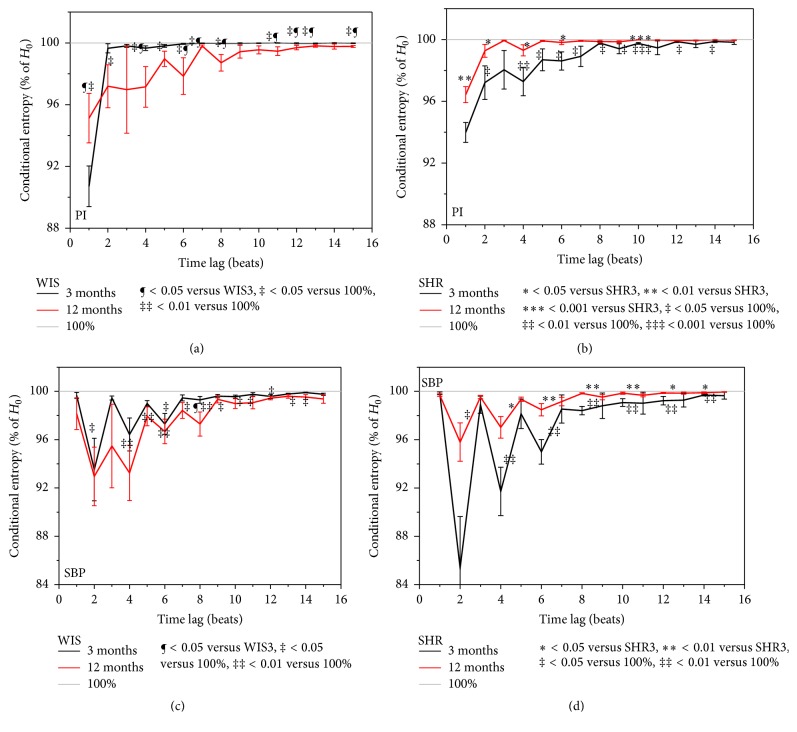
Relative conditional entropy estimates from binary differentially coded PI signals (a, b) and SBP signal (c, d).

Conditional entropy defined over the binary differentially coded signals (6) operates over coarsely coded signals, but it is computationally less demanding. The relative conditional entropy of signals taken from the same time series is presented in Figure 7.

Except for the time lag *α* = 1 (to be explained later on), conditional entropy of PI signals in young healthy Wistar rats is equal to its statistically independent counterpart. As the age increases, the PI signals exhibit more order (inputs are attenuated) and more mutual dependency so the entropy slightly decreases. Surprisingly, hypertensive rats (b, d) showed just opposite results: conditional and unconditional entropies were equal in aging rats, while young ones had a slight increase of statistical dependency between the signal samples at the time lag *α*, and the corresponding entropy was slightly lower. Considering the binary samples of SBP signals (c, d), the discrepancy of conditional entropy with respect to its unconditional counterpart is considerably enlarged. This discrepancy diminishes at time lag *α* = 10 (12 in hypertensive young rats); that is, there is no statistical dependency between the changes in blood pressure if the observed samples are at time lag of 10 (12) beats one from another.

**Figure 8:**
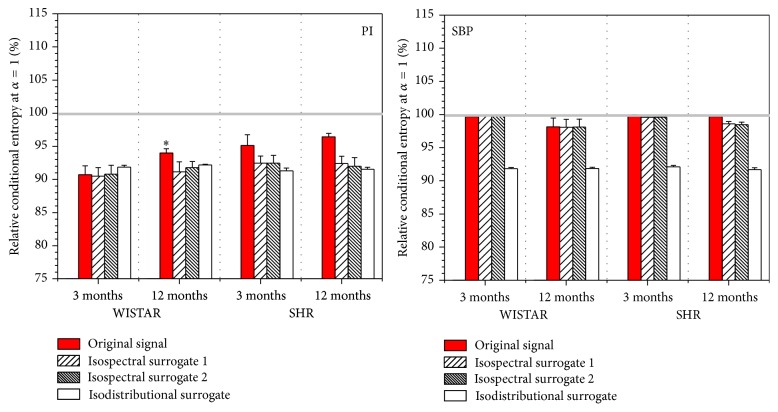
Relative conditional entropy estimated at time lag *α* = 1 from the original PI signals, original SBP signals, and the three types of surrogate control signals. The results are expressed as mean ± s.e.m; *∗* < 0.05 versus 3-month-old animals. Gray line shows the value of unconditional entropy.

The dependency at the time lag of *α* = 1 (adjacent symbols) is different as it is not related to the physiological constraints of PI and SBP signals. It is predominantly a consequence of the differential coding procedure: the adjacent samples of differentially coded signal both comprise the same original signal sample *x*_*i*+1_ (5). In the first differential sample *x*_*i*+1_ is a minuend, *xD*_*i*_ = *x*_*i*+1_ − *x*_*i*_, and in the second differential sample *x*_*i*+1_ is a subtrahend, *xD*_*i*+1_ = *x*_*i*+2_ − *x*_*i*+1_. The conditional entropy at time lag *α* = 1 is shown in Figure 8, including the entropy estimates of real signals (red bars) and, as a control, estimates taken from the surrogate signals. Isodistributional surrogates randomize the order of the original signal samples, thus destroying their dependency but preserving the distribution function. Isospectral surrogates randomize the signal phase thus altering the samples and their distribution, but preserving the spectral density, autocorrelation function, and, consequently, intersample relationship.

**Figure 9:**
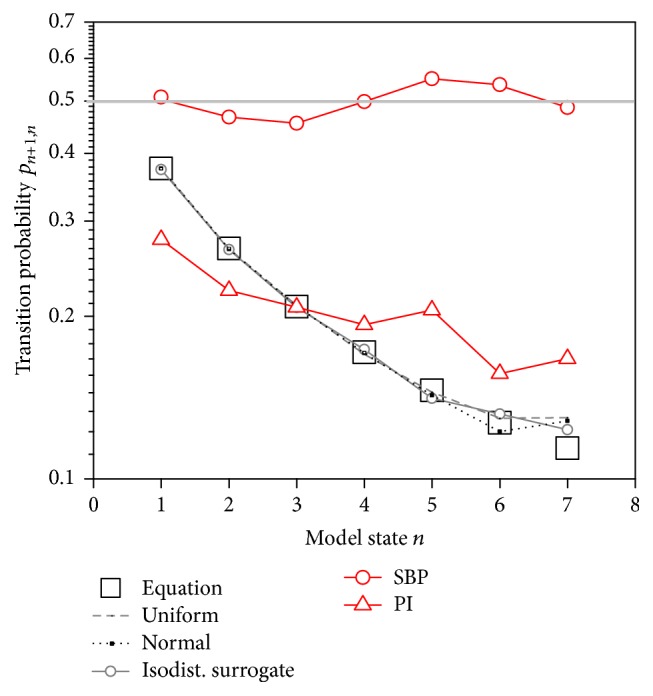
Transition probabilities of binary differentially coded signal samples (Figure 5), estimated from all the subjects. SBP probabilities are in a vicinity of 0.5. Probabilities estimated from random and randomized signals are in an excellent accordance with the probability in (8). PI probabilities differ from the previous groups.

Even if the signals are random and independent, their differentially coded counterparts are not. Differential coding forces the conditional entropy estimates of randomized data (isodistributional surrogates) to lose 8 to 9% of their values (white bars in Figure 8). These simulation results are in a perfect accordance with theoretical entropy loss that is equal to 8.17%, as shown in Appendix. Conditional entropy of PI signal and all of its control surrogates follow the theoretical constraints induced by differential coding. The same holds for isodistributional controls of SBP signals, since the random scrambling destroys intersample relationship. But SBP signals seems to be resilient to the coding-induced dependency, preserving the entropy value that, according to the theory, should be reserved for statistically independent binary data. The same applies to SBP Isospectral surrogates, since the phase randomization does not affect the intersample relationship. Seemingly, the regulatory mechanisms of systolic blood pressure are so firm and manage to oppose coding-induced dependency so well that the conditional entropy does not differ from its unconditional counterpart. It is also in accordance with the finding that the transition probabilities of differentially coded SBP samples (model in Figure 5) follow the Bernoulli distribution: the probabilities that the next SBP sample amplitude would increase or decrease are the same; that is, the transition probabilities for the model in Figure 5 are equal to *p*_*n*+1,*n*_ = 0.5 (Figure 9).

### 3.2. Dynamic Measures

**Figure 10:**
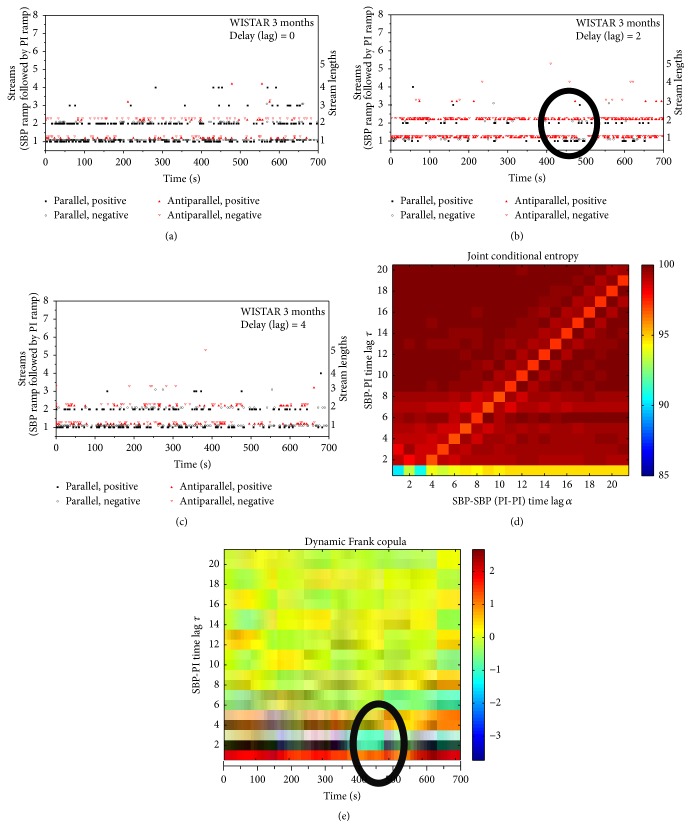
Dynamic observation of streams and copula; (a, b, c) show the position of a particular stream at the time axis; length of sequence is marked in the right, while the type of sequence is marked by a different symbol and a slight amplitude offset. (d) shows joint relative cross-entropy for different time lags *τ* and *α*, while (e) shows a copula plot, change of copula along the time axis.

Dynamic observation of copulas imply, as already said, an analysis of the overlapping segments of data and plotting the results obtained from each particular segment along the time axis (Figure 10(e)), keeping time lag *τ* as a parameter along the ordinate.

The changes in copula parameter might be a consequence of the appearance of parallel and antiparallel streams, so the occurrence of streams is plotted along the same time axis. (Figures 10(a), 10(b), and 10(c)). The plot distinguishes length of streams, marked by the corresponding amplitude in plot. Type of the stream, parallel and antiparallel, is marked black and red, respectively.

For time lags *τ* = 0 and *τ* = 4 parallel streams (black) are dominant (a) and (c). For time lag *τ* = 4, antiparallel streams (red (b)) outnumber the parallel ones. These observations are in a perfect accordance with the dynamic copula parameter in (e): at the time lags *τ* = 0 and *τ* = 4 dependency is expressed as a horizontal red line along the time axis, showing a strong positive dependence; at the time lag *τ* = 2, the dependence is negative (horizontal dark blue line). A temporary increased concentration of parallel streams between the seconds 400 and 500 (encircled region at *τ* = 2, (b)) is reflected in short drop of dependency strength marked with lighter blue color of short duration, encircled in (e).

Conditional entropy is estimated from a coarsely coded binary signal. Yet, as a method, it distinguishes the changes in entropy values at the time lags *τ*, shown by light red horizontal stripes in (d). The entropy changes have lower dynamic range as the coding itself is coarse, reducing 4096 levels of the original data to binary symbols. However, joint conditional entropy suffers a methodical drawbacks when the results are presented simultaneously in *α*-*τ* plane: levels of freedom *α* and *τ* shown in Figure 3 enable a deterministic sample overlap and induce a dependence that result in diagonal artifacts in Figure 10(d), that may cause an ambiguity in results.

Unconditional entropy as defined in the upper part of (8) corresponds to Shannon entropy JSD_Sh_ with *m* = 1, defined within the concept of joint symbolic dynamics (JSD) [46, 47]. JSD forms joint “words” taking *m* bits from each one of the observed time series, and, among the other parameters, it calculates unconditional entropy JSD_Sh_. Typically, *m* is equal to 3, so the cardinality of words is equal to 64, making conditional entropy difficult to achieve word-by-word estimation of the required 4096 transition probabilities, which is not suited with the concept of limited power resources that are the reason for including the binary operations [48].

**Figure 11:**
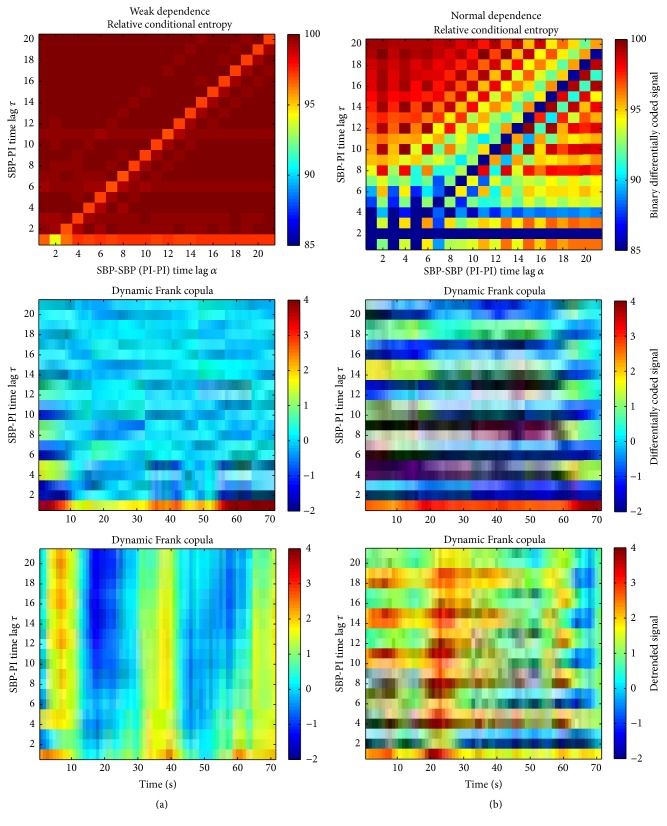
Joint conditional entropy and copula parameter observed along the time axis, (a) signals with poor statistical dependency; (b) signals with strong dependency (horizontal lines).

Figure 11 illustrates the characteristic cases. (a) corresponds to signals without exposed statistical dependency. Except for the unwanted but deterministic diagonal artifacts, increase of dependency in *α*-*τ* plane is registered only at *τ* = 1, for the adjacent SBP-PI pairs only, and the same applies for copula parameter estimated from differentially coded signals. Copula estimated from the raw signals, however, changes along the time axis. (b) corresponds to signals with strong statistical dependency. Entropy in *α*-*τ* plane, although with visible horizontal and vertical lines (the changes of dependency due to lags *τ* and *α*, resp.), also exhibits too many diagonal artifacts that make the image difficult to interpret. Dependency estimated by copula, from both raw data and differentially coded data, exhibits strong comonotonic and counter-monotonic relation at the characteristic time lags, shown by dark red and dark blue horizontal lines. Copulas also reveal an interesting phenomenon: statistical dependency can decrease, change the sign, or completely vanish along the time axis (middle and especially lower panels in Figure 11). That might point out a short temporary loss of these portions of neural connection that can be measured by SBP-PI interdependency.

To explore the relationship between the copula parameters and streams, the number of parallel and antiparallel streams at different time lags is shown in Table 2. Antiparallel streams are considered as “increasing” if SBP samples increase and PI samples decrease.

**Figure 12:**
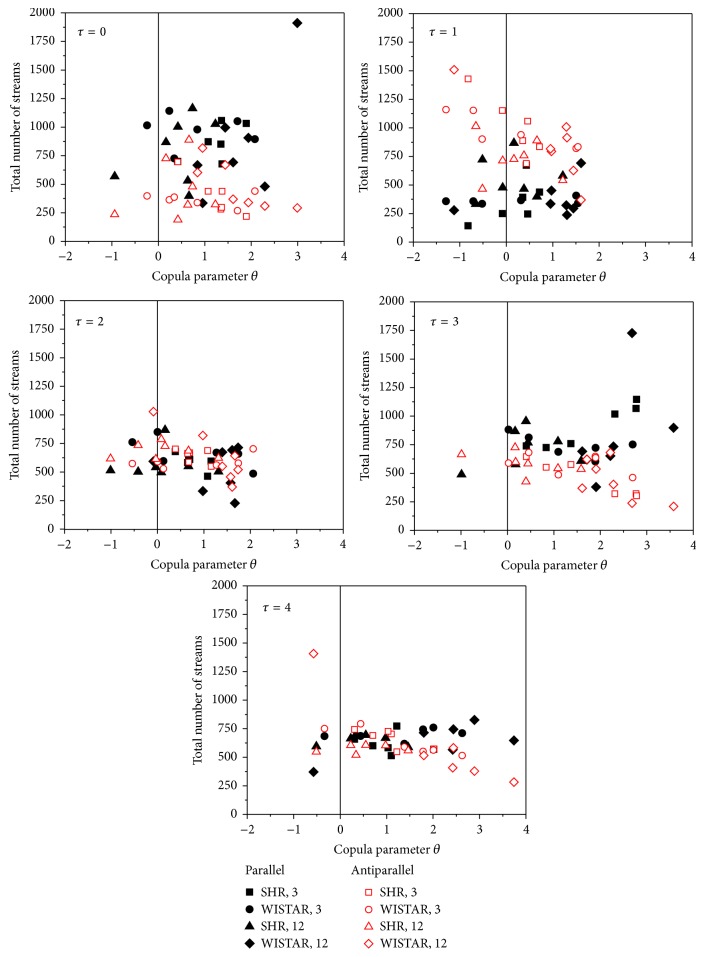
Number of parallel streams (black) and antiparallel streams (red) plotted against the copula parameter *θ*. Each point is estimated from 4000 SBP-PI signal samples.

The statistically significant differences exist between the numbers of parallel and antiparallel streams, but not between the different groups of animals. The average number of parallel streams is extremely small at time lags *τ* = 1 and 5, while it is extremely large for time lag *τ* = 0. It is in accordance with the mean copula values shown in Figure 6. The streams are further connected with copula parameters in Figure 12: for each rat a copula parameter is estimated and the number of parallel and antiparallel streams are counted. The *x*-axis of the obtained plots presents a copula parameter, while the *y*-axis presents the number of streams. A visual inspection of plots reveals that the same number of parallel and antiparallel streams in one rat produce positive dependence with *θ* = 1 and in the other rat negative dependence with *θ* = −1. Therefore, the copula value is related to the number of parallel and antiparallel streams, but loosely, and the dependency that copula reveals is more complex to be explained by the occurrences of SBP and PI ramps that change their amplitudes in the same or in the opposite direction.

## 4. Conclusion

The aim of this paper was to explore a time lag along which a change in one signal sample affects the changes of other samples in rats with hypertension and with increased age. Healthy Wistar rats, young and aging, were used as control. The dependency is measured using copula method at signal level (both raw signal and differentially coded signal) and at the binary level (binary differentially coded signals). Tools for assessing dependency were copulas within the field of real numbers and conditional entropy within the binary field.

Copulas applied as a static measure of SBP-PI dependency showed that the highest level of comonotonic behavior of PI with respect to SBP is observed at time lags *τ* = 0, 3, and 4, while a strong counter-monotonic behavior occurs at time lags *τ* = 1 and 2 in all four animal groups and is observable both for raw and for differentially coded signals. Dynamic range of copula parameter in aging rats was considerably reduced in hypertensive groups, showing a reduced capability for SBP-PI responses along the time axis. Contrary to this, dynamic range in healthy rats remained intact. Copula parameter observed along the time axis can be loosely related to the number of parallel and antiparallel streams and, indeed, the time lags with considerably increased average number of parallel streams correspond to the time lags that exhibits the strongest (averaged) comonotonic dependence and vice versa, the lags with increased number of antiparallel streams are the ones with lowest copula parameter. The number of streams has not shown statistically significant difference among the experimental groups but did show the difference at different time lags. When the copula parameter is related to the number of streams for each rat separately, it turned out that the same number of parallel and antiparallel streams produced positive dependency in one rat and negative dependency in another rat for the same time lag. It shows that the dependency that copula reveals is more complex to be explained by mere occurrences of parallel and antiparallel SBP-PI streams. This conclusion is also confirmed with decreased dynamic range of copula parameter: it was considerably attenuated in hypertensive rat with an increased age, although no significant change in number of streams is observed.

Conditional entropy is a measure applicable to the binary data, important for applications in wearable battery-operated devices (crowdsensing, self-monitoring), where saving the processor power and increased computing efficiency are the ultimate goal. Although the binary coding is extremely coarse, conditional entropy can observe the changes in sample dependency. The sensitivity is slightly reduced, due to the reduced number of amplitude levels of the observed signals. Conditional and unconditional entropy of PI signals in young healthy Wistar rats are equal, revealing the sample independence. As the age increases, the PI signals exhibit more order (inputs are attenuated) and more mutual dependency so the entropy slightly decreases. Surprisingly, hypertensive rats showed just opposite results: conditional and unconditional entropies were equal in aging rats, while young ones had a slight increase of statistical dependency. Conditional entropy of SBP signals shows a considerable discrepancy with respect to unconditional counterparts that diminishes at time lag *α* = 12 in hypertensive young rats and at time lag *α* = 10 in all the other groups. Simultaneous observation of entropy changes in *τ*-*α* plane is not recommended, as the artifacts due to the signal overlap occur. The level of conditional entropy at time lag *α* = 1 (adjacent symbols) is reduced by a theoretical value of 8.17%, induced by the constraints of differential coding. This applies if the raw data are random and statistically independent, and this also applies to PI signals, to their isospectral surrogates, and to isodistributional surrogates of all the signals. SBP signals, however, preserve the equality of conditional and unconditional entropy in spite of dependency induced by differential coding. Seemingly, the regulatory mechanisms of systolic blood pressure are so firm and manage to oppose coding-induced dependency.

Dynamic tracing the dependency parameters shows that, occasionally, SBP and PI signals may become unconnected. A future task would include quantification of these occurrences and mode of their exploitation. Another goal of the future research would be to include multivariable time series (respiratory rate, such as in [49], or temperature), since the copula method allows creation of dependency structures among multidimensional signals.

## Figures and Tables

**Table 1:** Mean and s.e.m. of measured time series, classical variability analysis.

	SBP [mmHg]	DBP [mmHg]	PI [ms]	Poincaré plot		X*ApEn*
				SD1 [ms]	SD2 [ms]	
SHR 3	172.04 ± 4.25	117.53 ± 4.93	184.13 ± 6.04	2.42 ± 0.28	11.01 ± 1.78	1.59 ± 0.10
SHR 12	200.47 ± 5.01^*∗∗*^	134.10 ± 2.59^*∗*^	197.05 ± 6.10	3.8 ± 0.97	14 ± 2.16	1.39 ± 0.17
WIS 3	123.99 ± 3.82	77.66 ± 4.32	174.02 ± 4.69	2.63 ± 0.37	10.1 ± 1.4	1.38 ± 0.08
WIS 12	132.81 ± 2.44	87.51 ± 1.14	174.18 ± 3.38	3.15 ± 0.45	11.09 ± 1.64	1.49 ± 0.12

Data are expressed as mean ± s.e.m. *∗* < 0.05 versus SHR3. *∗∗* < 0.01 versus SHR3.

**Table 2:** Mean number of streams ± s.e.m. at different SBB-PI time lags *τ*. Streams have two attributes: parallel or antiparallel, positive or negative.

	Parallel+	Parallel−	Antipar+	Antipar−	Parallel+	Parallel−	Antipar+	Antipar−
	Lag *τ* = 0				Lag *τ* = 1			
SHR 3	213.16 ± 34.27	227.33 ± 28.67	70.33 ± 18.98	67 ± 21.11	74.16 ± 24.88	62.83 ± 22.12	276.66 ± 50	356.16 ± 62.89
SHR 12	268.33 ± 43.27	241.16 ± 40.46	86.66 ± 19.66	79.15 ± 12.81	120 ± 23.87	112 ± 13.55	157 ± 30.79	211.66 ± 34.06
WIS 3	268.833 ± 25.44	277.83 ± 24.96	62.5 ± 6.42	74.5 ± 5.89	63.66 ± 7.16	54.3 ± 6.95	238 ± 33.78	289.333 ± 24.02
WIS 12	288.66 ± 87.92	286.33 ± 86.19	57 ± 15/3	70 ± 12.8	59.33 ± 13.98	61.666 ± 8.3	245.66 ± 48.85	302.833 ± 30.35

	Lag *τ* = 2				Lag *τ* = 3			
SHR 3	121.5 ± 12.59	129.5 ± 12	130.83 ± 14.35	136.16 ± 15.46	306.66 ± 42.42	233 ± 25.9	76.16 ± 19,97	108.33 ± 20.17
SHR 12	109.83 ± 3.42	121.66 ± 6.24	156 ± 13.1	165.83 ± 4.27	211.16 ± 28.07	176 ± 25.77	121.16 ± 9,06	135.33 ± 10.21
WIS 3	155 ± 15.08	169.166 ± 18,99	122.33 ± 10.97	131.5 ± 11.72	212.16 ± 18.19	181.33 ± 16.05	101.5 ± 14.42	140.83 ± 12.46
WIS 12	111.5 ± 18.21	96.333 ± 26.86	156 ± 25.35	172.66 ± 24.28	275.66 ± 72.40	227.333 ± 74.06	88 ± 17.14	112.66 ± 25.24

	Lag *τ* = 4				Lag *τ* = 5			
SHR 3	144.16 ± 15.09	131.33 ± 13.12	159.16 ± 16.73	155.16 ± 10.89	98 ± 22.63	100.16 ± 20.30	195.83 ± 23.51	236.16 ± 26.38
SHR 12	170.83 ± 19,82	155.33 ± 10.88	119.83 ± 2.85	132.16 ± 4.222	141.5 ± 15,12	134.5 ± 6.13	145.833 ± 15.89	174.33 ± 18,49
WIS 3	191.66 ± 11.68	175.333 ± 5.85	126.83 ± 19.91	152.333 ± 13.60	119.5 ± 15.18	127.16 ± 21.23	151.166 ± 12.22	193.83 ± 13.95
WIS 12	187.33 ± 32,59	164 ± 23.96	137.5 ± 59.07	150.66 ± 53.56	131.5 ± 20.76	121.833 ± 17.64	153.16 ± 22	195.5 ± 30.81

## Appendix

The purpose of this appendix is to derive an exact amount of conditional entropy loss if a random and statistically independent signal is submitted to the binary differential coding.

The probability that the adjacent binary differentially coded samples would be both equal to zero is equal to

(A.1) PxBi=1,xBi+1=1=PxDi<0,xDi+1<0=Pxi+1−xi<0,xi+2−xi+1<0.

The notation is taken from (5), (6), and (7).

If *x*_*i*_ are independent and identically distributed random variables (i.i.d. RVs) with probability distribution function *f*(*x*_*i*_), then the probability (A.1) can be obtained as follows:

(A.2)

Similarly,

(A.3) PxBi=0,xBi+1=0=∫−∞∞fxi+1·∫xi+1∞fxi+2·dxi+2·∫−∞xi+1fxi·dxi·dxi+1,PxBi=0,xBi+1=1=∫−∞∞fxi+1·∫−∞xi+1fxi+2·dxi+2·∫−∞xi+1fxi·dxi·dxi+1,PxBi=1,xBi+1=0=∫−∞∞fxi+1·∫xi+1∞fxi+2·dxi+2·∫xi+1∞fxi·dxi·dxi+1,PxBi=0=∫−∞∞fxi+1·∫−∞xi+1fxi·dxi·dxi+1,PxBi=1=∫−∞∞fxi+1·∫xi+1∞fxi·dxi·dxi+1.

For i.i.d. RVs with uniform probability density function *f*(*x*) = 1/*a*, 0 ≤ *x* ≤ *a*, and *f*(*x*) = 0 elsewhere, the probabilities defined with (A.3) are equal to *P*(00) = *P*(11) = 1/3, *P*(10) = *P*(01) = 1/6, and *P*(0) = *P*(1) = 0.5. Conditional probabilities that follow are *P*(0∣0) = *P*(1∣1) = 2/3 and *P*(1∣0) = *P*(0∣1) = 1/3. All four conditional probabilities in statistically independent data are equal to 0.5. Implementing (7), lower part, entropy values (conditional and unconditional) are 0.27643 and 0.30103, while their relative measure is 91.92968, perfectly in accordance with the values estimated from randomized signal (isodistributional surrogate data). This conclusion is general, since it was proven [30] that the differentially coded samples of i.i.d. RVs (including the isodistributional surrogates) statistically follow the same distribution.
